# A role for the histone deacetylase HDAC4 in the life-cycle of HIV-1-based vectors

**DOI:** 10.1186/1743-422X-7-237

**Published:** 2010-09-16

**Authors:** Johanna A Smith, Jennifer Yeung, Gary D Kao, René Daniel

**Affiliations:** 1 Division of Infectious Diseases - Center for Human Virology, Department of Medicine, Thomas Jefferson University, Philadelphia, PA 19107, USA; 2 Department of Radiation Oncology, University of Pennsylvania School of Medicine, Philadelphia, PA 19104, USA; 3 Center for Stem Cell Biology and Regenerative Medicine, Thomas Jefferson University, Philadelphia, PA 19107, USA; 4 Kimmel Cancer Center, Immunology Program, Thomas Jefferson University, Philadelphia, PA 19107, USA

## Abstract

HIV-1 integration is mediated by the HIV-1 integrase protein, which joins 3'-ends of viral DNA to host cell DNA. To complete the integration process, HIV-1 DNA has to be joined to host cell DNA also at the 5'-ends. This process is called post-integration repair (PIR). Integration and PIR involve a number of cellular co-factors. These proteins exhibit different degrees of involvement in integration and/or PIR. Some are required for efficient integration or PIR. On the other hand, some reduce the efficiency of integration. Finally, some are involved in integration site selection. We have studied the role of the histone deacetylase HDAC4 in these processes. HDAC4 was demonstrated to play a role in both cellular double-strand DNA break repair and transcriptional regulation. We observed that HDAC4 associates with viral DNA in an integrase-dependent manner. Moreover, infection with HIV-1-based vectors induces foci of the HDAC4 protein. The related histone deacetylases, HDAC2 and HDAC6, failed to associate with viral DNA after infection. These data suggest that HDAC4 accumulates at integration sites. Finally, overexpression studies with HDAC4 mutants suggest that HDAC4 may be required for efficient transduction by HIV-1-based vectors in cells that are deficient in other DNA repair proteins. We conclude that HDAC4 is likely involved in PIR.

## Introduction

Chromatin undergoes expansion and compaction in the course of many fundamental cellular processes, including gene expression, differentiation, cell cycle progression and DNA repair. These alterations of the chromatin structure are largely mediated by histone acetylases and histone deacetylases (HDACs). HDACs deacetylate key lysine residues of core histones to induce chromatin compaction. This process usually results in transcriptional repression [1]. Cells contain many HDACs, which are categorized into four classes, based on sequence homologies. Class I (homologues of the yeast deacetylase Rpd3) contains HDAC1, HDAC2, HDAC3 and HDAC8 [2-6]. Class II (yeast Hda1 homologues) contains HDAC4, HDAC5, HDAC6 and HDAC7 [7-12]. Class II HDACs, unlike Class I, can shuttle in and out of the nucleus, depending on various signals [13]. Class III contains proteins that are homologous to the yeast deacetylase Sir 2 [14,15]. Finally, the Class IV contains enzymes which are related to those of Class I and Class II, but a sequence analysis shows they form a distinct class. They are exemplified by HDAC11 [16].

Although transcriptional repression is apparently an important function of HDACs, these proteins seem to play a broader role in regulating cellular processes and one HDAC, HDAC4, has been found to play a role in cellular double-strand DNA break (DSB) repair. It has been shown by Kao et al. (2003) that HDAC4 forms nuclear foci in cells exposed to ionizing radiation, which causes double-strand DNA breaks [17]. Foci of DNA repair proteins are formed at sites of double-strand DNA breaks, and the HDAC4 foci overlap with foci of the DNA repair proteins Rad51 and 53BP1. Silencing of HDAC4 via RNA interference leads to radiosensitisation of HeLa cells, underscoring a requirement for HDAC4 in DSB repair. In addition, HDAC4-deficient cells were shown to loose the DNA damage-induced G2/M checkpoint. The molecular function of HDAC4 in DSB repair remains to be fully clarified, although it has been shown very recently that nuclear translocation of HDAC4 is required and it may play a role in the suppression of promoters of genes that are activated during G2/M progression [18,19].

It has been shown previously by us and others that cellular DSB repair proteins are involved in the life-cycle of retroviruses and retroviral vectors. We have observed that cellular DSB proteins are involved in completing the integration process. In addition, others suggested that they are involved in the formation of 2-LTR circles, and it has been proposed that they might also be involved in intranuclear trafficking of the preintegration complex [20-23].

In this study, we have tested the hypothesis that HDAC4 plays a role in the life-cycle of HIV-1-based vectors. We show that infection with retroviral vectors induces, similar to DSBs, nuclear foci of the HDAC4 protein. We show that the formation of these foci is dependent on active retroviral integrase, and HDAC4, but not HDAC2 and HDAC6, associates with viral DNA. Taken together, these data indicate that HDAC4 plays a yet undiscovered role at sites of retroviral DNA integration. In addition, we show that overexpression of nuclear HDAC4 rescues a defect in retroviral transduction that is associated with a deficiency of the cellular DNA repair protein ATM. We conclude that HDAC4 is involved in stable transduction by retroviral vectors, and plays a role in the completion of the integration process.

## Results

### HDAC4, but not HDAC2 or HDAC6, associates with DNA of an infecting HIV-1-based vector

**Figure 1:**
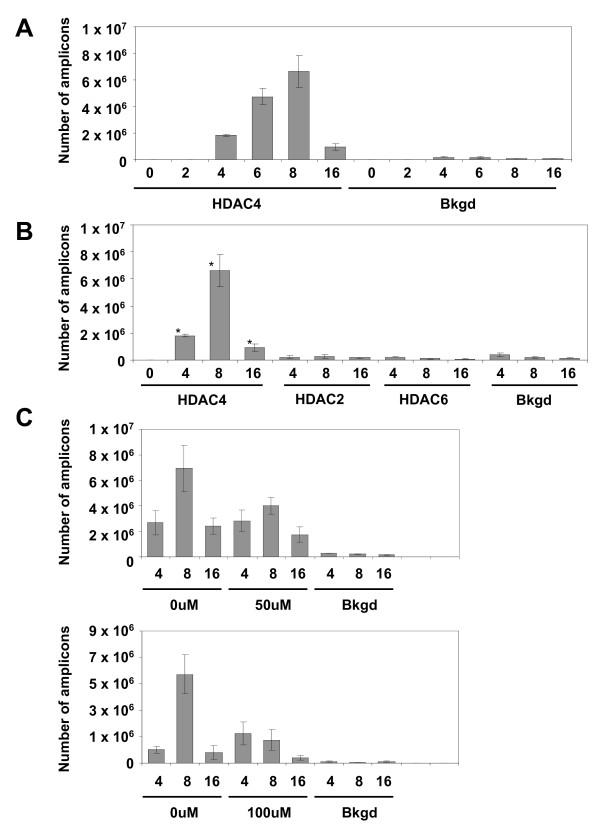
**HDAC4 associates with vector DNA in an integrase-dependent manner**. **(A) **ChIP analysis of infected HeLa cells. To establish if HDAC4 associates with vector DNA, HeLa cells were infected with the HIV-1-based vector at an m.o.i. of 0.1 and ChIP was performed with the anti-HDAC4 antibody as described in "Experimental Procedures", followed by real-time PCR to detect vector DNA. Numbers (x-axis) indicate hours post-infection. Bkgd - background, no antibody added. **(B) **HDAC2 and HDAC6 association with vector DNA. Lysates of cells infected as described above (Fig. 1A) were immunoprecipitated with antibodies against HDAC2 and HDAC6, as indicated. Terminology as above, * indicates samples from A. **(C) **Effect of an integrase inhibitor on the association of HDAC4 with vector DNA. Cells were infected as in A, except the integrase inhibitor 118-D-24 was added to samples at the indicated concentrations, together with the vector. Cells were processed as in A.

HeLa cells were infected with a pseudotyped HIV-1-based vector (containing a *lac*Z reporter) at an m.o.i. of 0.1 and harvested at the time points indicated (Fig. 1A). Chromatin immunoprecipitation (ChIP) analysis was used to identify the association of HDAC4 with viral DNA. To do so, DNA isolated from infected cells and the associated proteins were crosslinked, immunoprecipitated with the HDAC4 antibody (see Methods), and associated viral DNA was amplified by real time PCR. Results are expressed as a number of viral DNA amplicons per μl of chromatin immunoprecipitates at each time point. As shown in Fig. 1A, viral DNA was found to be associated with HDAC4 at 4, 6, 8, and 16 hrs post-infection. The amount of HDAC4-associated viral DNA steadily increased from 4 hours, with a peak reached at 8 hours post-infection. The associated viral DNA drastically declined at the 16 hour time point. To determine if vector DNA associates with other HDAC proteins, we have immunoprecipitated lysates from infected cells with the HDAC2 and HDAC6 antibodies. Whereas HDAC2 is a Class I HDAC, we note HDAC6 is a class II HDAC and thus structurally closely related to HDAC4. However, as shown in Fig. 1B, we did not observe any association of these HDACs with viral DNA. We thus conclude that HDAC4 shows a distinct preference for association with vector DNA, when compared to other HDACs.

### Retroviral integration enhances the association of HDAC4 with vector DNA

We note that HDAC4 was reported to associate with DNA of the avian sarcoma virus, but this association was detected only post-integration [24]. To test the hypothesis that integration is required for the association of HDAC4 with the DNA of HIV-1-based vectors, we have infected HeLa cells and treated them with the integrase inhibitor 118-D-24. As shown in Fig. 1C, the inhibitor decreases the association of HDAC4 with vector DNA in a dose-dependent manner. However, we note that the inhibitor effect can be seen only at 8 hours post-infection, when the association of DNA with HDAC4 is at its peak. In contrast, association at 4 hours post-infection is resistant to the inhibitor treatment. These data suggest that while integration does stimulate the association of vector DNA with HDAC4, HDAC4 also associates with vector DNA prior to integration, in an integration-independent manner.

### Retroviral integration induces the formation of HDAC4 foci in infected cells

**Figure 2:**
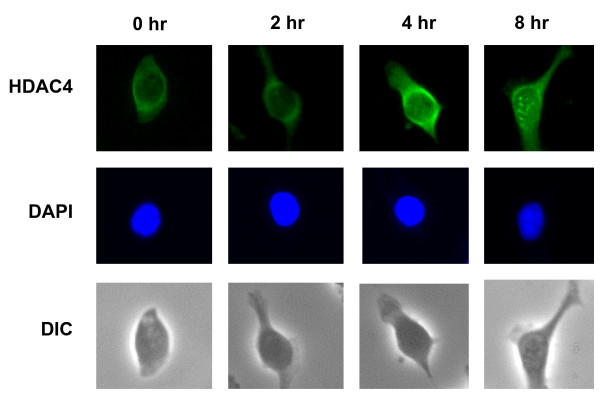
**The HDAC4 protein forms foci following infection with the HIV-1-based vector**. HeLa cells were infected with an HIV-1-based vector as described in "Experimental Procedures". At indicated time points, samples were fixed and stained with the anti-HDAC4 antibody (top row). Nuclei were visualized using DAPI staining (middle row). Representative photographs are shown. DIC (differential interference contrast) shows the cell morphology (bottom row).

**Figure 3:**
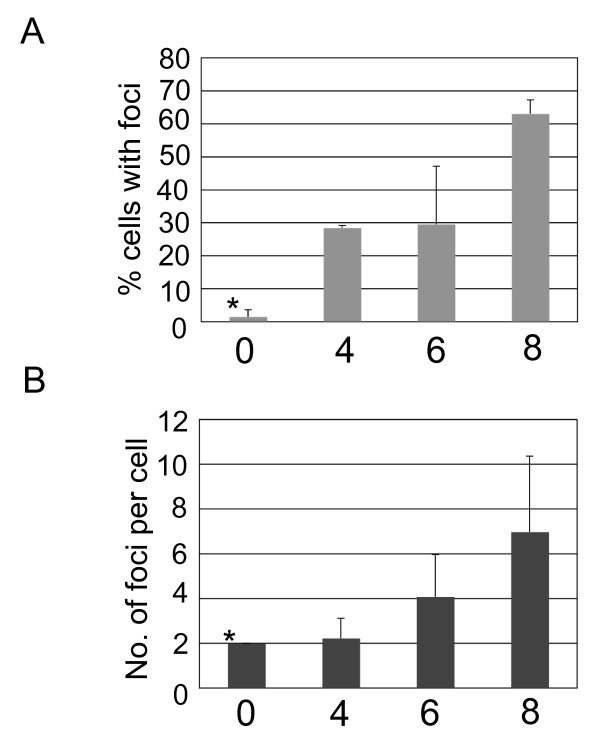
**Quantitative analysis of HDAC4 foci in the vector-infected cells**. Number of HeLa cells containing foci, as well as foci number per cells were counted in images prepared as described in Fig. 2 and "Experimental Procedures". (**A) **Number of foci-containing cells. (**B) **Average number of foci among foci-containing cells. Numbers (x axis) indicate hours post-infection. Bars indicate standard deviation. * - only one foci-containing cell was found.

HDAC4 was reported to form foci in irradiated cells. These foci were associated with the formation of double-strand DNA breaks [17]. To determine if infection with HIV-1-based vectors induces the formation of HDAC4 foci, we have infected HeLa cells at a high multiplicity of infection (10), fixed infected cells at predetermined time points and stained with the HDAC4 antibody. We observed that in uninfected cells, HDAC4 is present almost exclusively in the cytoplasm (Fig. 2). Similarly, we have observed that HDAC4 is predominantly cytoplasmic at 4 and 6 hours post-infection. However, we also observed the appearance of HDAC4 foci in infected cells, with the majority of cells containing foci at 8 hrs post-infection (Figs. 2 and 3). Most of the infected cells contained multiple HDAC4 foci. As indicated in Fig. 3, the number of foci correlates well with the multiplicity of infection.

**Figure 4:**
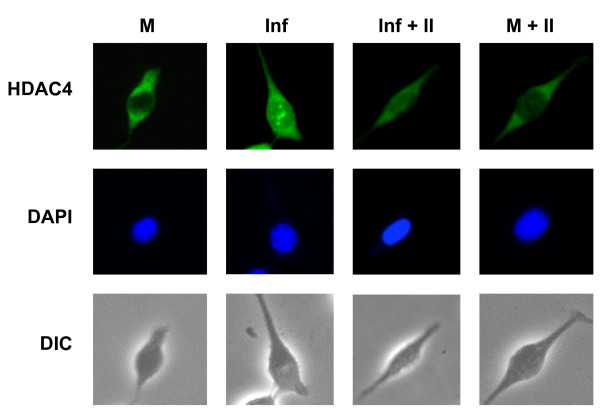
**Effect of an integrase inhibitor on the formation of HDAC4 foci in infected cells**. HeLa cells were infected with the HIV-1-based vector in the presence and absence of the integrase inhibitor (100 μM), or were treated only with the inhibitor, as indicated. Cells were processed as in Fig. 2, at 8 hrs post-infection. M - mock, uninfected cells, Inf - cells infected with the HIV-1-based vector, Inf+II - cells infected with the HIV-1-based vector and treated with the integrase inhibitor (added together with the virus), II - cells treated with the integrase inhibitor only. Other terminology as in Fig. 2.

**Figure 5:**
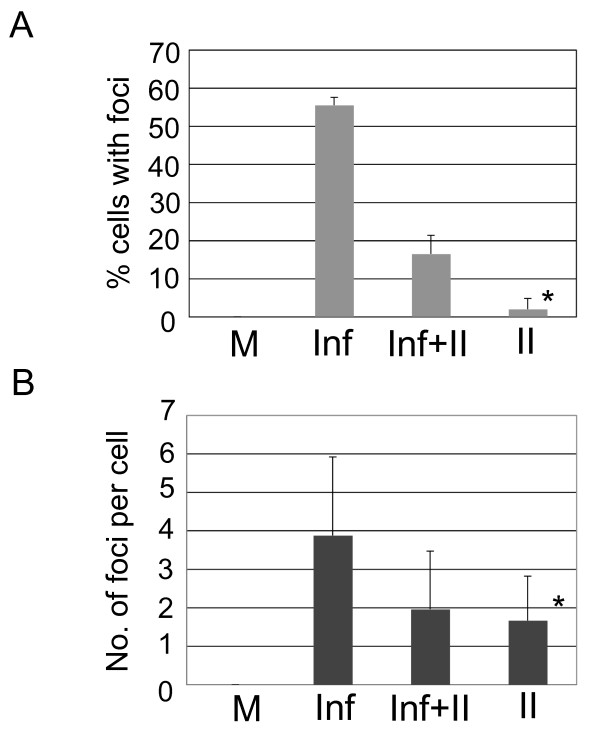
**Quantitative analysis of HDAC4 foci in cells infected with the HIV-1-based vector and treated with an integrase inhibitor**. Samples from Fig. 4 were quantitated as described in Fig. 3 and "Experimental Procedures". (**A) **Number of foci-containing cells. (**B) **Average number of foci among foci-containing cells. Bars indicate standard deviation. * - only three foci-containing cells were found. DIC - phase contrast.

We have observed that integration stimulates the association of HDAC4 with vector DNA and wondered if integration affects the formation of HDAC4 foci. Thus, we have infected HeLa cells in the presence and absence of an integrase inhibitor. We have again detected HDAC4 foci in cells that were infected with the HIV-1-based vector in the absence of the inhibitor (Fig. 4). However, treatment of infected cells with the inhibitor significantly reduced (ca. 3.5 fold) the total number of cells that contained foci (Fig. 4 and Fig. 5). We have also observed a drop in the average number of foci per cell among foci-containing cells, although the difference was within the standard deviation due to a wide range of the numbers of foci (1 to 8 foci per cell among cells infected with the vector and 1 to 6 foci per cell in cells infected with the vector and treated with the inhibitor, Fig. 5B). Treatment with the inhibitor itself had a negligible effect on the intracellular localization of HDAC4 (Figs. 4 and 5). Taken together, our results suggest that although HDAC4 associates with viral DNA even prior to integration, integration stimulates further accumulation of HDAC4 at integration sites, which are then marked by the formation of HDAC4 foci.

### Effect of HDAC4 knockdown on HIV-1 transduction

**Figure 6:**
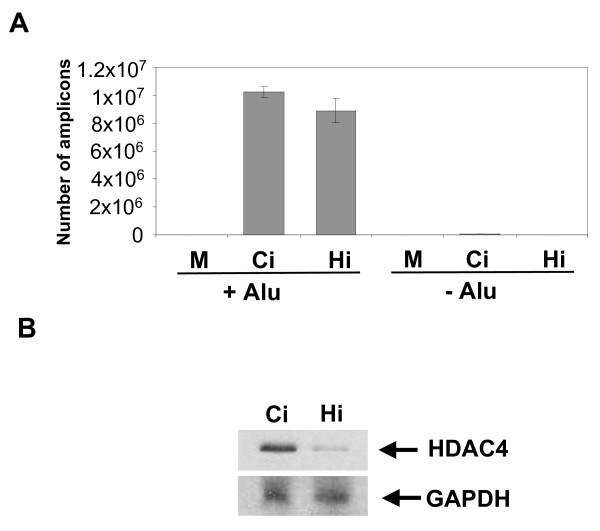
**Effect of HDAC4 knockdown on the efficiency of integration and PIR**. **(A) **HDAC4 was suppressed in HeLa cells using an siRNA treatment two days in a row (see "Experimental Procedures"). Two days after the first siRNA transfection, cells were infected with the HIV-1- based vector. DNA was extracted 3 days post-infection and analyzed by *Alu*-PCR (see "Experimental Procedures"). +Alu - DNA was analyzed using *Alu*-PCR, -Alu - a negative control, the Alu primer was left out in the first round of PCR. M - uninfected cells, Ci - cells transfected with control siRNA and infected with the vector, Hi - cells transfected with HDAC4 siRNA and infected with the vector. **(B) **HDAC4 levels in cells transfected with control (Ci) and HDAC4 siRNA (Hi).

We have established a novel interaction between the cellular HDAC4 protein and HIV-1-based vectors. Our results suggested that HDAC4 plays a role in the life-cycle of these vectors. To test this hypothesis, we have knocked down HDAC4 in HeLa cells using siRNA treatment and determined if HDAC4 is required for stable integration of HIV-1- vector DNA. As shown in Fig. 6, HDAC4 had little effect on the efficiency of integration as measured by *Alu*-PCR. In addition, we have infected siRNA-treated cells with the HIV-1-based vector carrying an EGFP marker and examined EGFP expression using flow cytometry. We have not observed a significant drop in EGFP expression in HDAC4 siRNA-treated cells (data not shown). We conclude that HDAC4 deficiency does not appear to significantly affect the efficiency of integration. Similarly, it appears that HDAC4 is not necessary for the last step of the integration process, termed post-integration repair (PIR), since PIR failure results in a loss of cells in which integrase-mediated joining occurred, and thus again manifests as a decrease in the *Alu*-PCR signal [25,26].

### HDAC4 is involved in PIR in ATM-deficient cells

**Figure 7:**
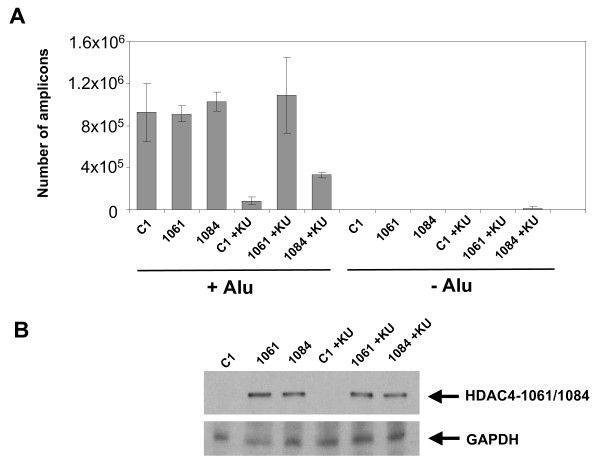
**Effects of overexpression of HDAC4 mutants on stable integration in ATM-deficient cells**. **(A) **Control HeLa cells and HeLa cells overexpressing HDAC4 mutants were infected and treated with the ATM inhibitor. One day post-infection, cells were harvested, DNA extracted and stable integration analyzed by *Alu*-PCR. C1 - cells transfected with the control EGFP expressing PEGFP-C1 plasmid and infected with the vector, 1061 - cells expressing the HDAC4-1061 mutant and infected with the vector, 1084 - cells expressing the HDAC4-1084 protein and infected with the vector. KU - the ATM inhibitor, KU-55933. +Alu - DNA was analyzed using *Alu*-PCR, -Alu - a negative control, the Alu primer was left out in the first round of PCR. **(B) **Comparison of the levels of overexpressed HDAC4 proteins. Western blotting was performed with an anti-GFP antibody (sc-9996, Santa Cruz), since HDAC4-1061 and HDAC4-1084 are fused to the GFP protein [29].

HDAC4 is a DSB repair protein, and it had been reported by us and others that these proteins are involved in PIR [23]. However, cellular DSB repair proteins often have overlapping functions and DSB repair systems can partially substitute for each other [27]. It is thus possible that a loss of the HDAC4 protein can be compensated for by other DSB repair systems or proteins. To test this hypothesis, we have induced a DSB repair deficiency in HeLa cells by treatment with an established ATM inhibitor, KU-55933 [28]. The ATM protein is a major player in cellular DSB repair and was reported by us and others to be involved in PIR [26-28]. At the same time, in these cells, we have overexpressed the HDAC4 protein. Since the normal HDAC4 protein (denoted here as HDAC4-1084) is mainly cytoplasmic, we have also overexpressed a mutant, which lacks a nuclear export signal (HDAC4-1061) and is thus present in the nucleus (Fig. 7, [29]). As expected, the ATM inhibitor reduced the *Alu*-PCR signal due to the inhibition of PIR (Fig. 7A). We have observed that in ATM-proficient HeLa cells, the overexpressed HDAC4 proteins do not appear to affect the efficiency of integration or PIR, as shown by *Alu*-PCR (Fig. 7A). However, in HeLa cells that were treated with the ATM inhibitor, the HDAC4-1084 reverses the inhibitor effect and upregulates HIV-1 transduction four fold. The HDAC4-1061 mutant that is constitutively present in the nucleus completely reverses the effect of the ATM inhibitor (Fig. 7A). To investigate the possibility that the differences in the *Alu*-PCR signals could be due to variations of the exogenous HDAC4 expression levels, we performed a western bloting analysis (Fig. 7B). However HDAC4-1061 and HDAC4-1084 levels appear to be the same in our transfected cells. Taken together our results suggest that HDAC4 is involved in PIR, but its function can be replaced by other DSB protein(s).

**Figure 8:**
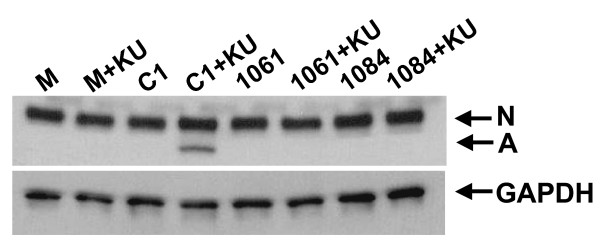
**Effects of overexpression of HDAC4 mutants on integrase-dependent apoptosis in ATM-deficient cells**. Control HeLa cells and HeLa cells overexpressing HDAC4 mutants were infected at m.o.i. 2 and treated with the ATM inhibitor (10 μM). One day post-infection, cells were harvested, lysed and cell lysates subjected to western blotting with an anti-PARP antibody. M - uninfected HeLa cells, C1 - cells transfected with the control EGFP expressing PEGFP-C1 plasmid and infected with the vector, 1061 - cells expressing the HDAC4-1061 mutant and infected with the vector, 1084 - cells expressing the HDAC4-1084 protein and infected with the vector. KU - the ATM inhibitor. N - normal PARP protein, A - the 85-kDa fragment of PARP, which is an apoptotic marker. GAPDH served as a loading control.

Finally, a failure of PIR induces apoptotic death of infected cells. If the effect of nuclear HDAC4 on infection efficiency is due its role in PIR, it should prevent PIR-associated cell death. Thus, cells were treated and infected as above (Fig. 7), except at a high m.o.i. (2), and analyzed by Western blotting for the presence of the 85-kDa PARP fragment, an apoptotic marker generated by caspase-mediated cleavage of the PARP protein [30]. As shown in Fig. 8, ATM inhibition and infection stimulated PARP cleavage. However, the apoptosis was reduced by overexpression of either the full length HDAC4 (HDAC4-1084) or the truncated mutant (HDAC4-1061). This finding is again consistent with an HDAC4 role in PIR.

## Discussion

In this study, we demonstrate that the histone deacetylase HDAC4, a Class II HDAC, associates with DNA of HIV-1-based vectors and forms foci at sites of integration. We also show that overexpression of nuclear HDAC4 rescues the defect in PIR that is induced by an ATM deficiency. Our data thus reveals a new cellular partner, which is involved in the life-cycle of HIV-1-based vectors. Our finding also supports the hypothesis that cellular DSB repair proteins are involved in PIR. At the same time, these proteins clearly have overlapping functions and can to a degree substitute for each other.

What could be the HDAC4 function in PIR? HDAC4 is a deacetylase, although with relatively low activity [31]. Histone deacetylation generally results in transcriptional suppression. Thus, one possible function of HDAC4 could be to suppress transcription at integration sites, thus allowing access for DNA repair machinery. We note in this context that HIV-1 prefers to integrate in genes and the likelihood of transcription interfering with integration is thus very high [32]. Second, it is possible that HDAC4 is required for the recruitment of other DNA repair proteins to PIR sites. HDAC4 was reported to physically interact with the 53BP1 protein and thus may bring this protein to PIR sites. It will be a matter of future experiments to distinguish between these possibilities.

We also note that HDAC4 associates with vector DNA prior to integration. These data suggest that HDAC4 may play a role in steps prior to PIR. However, since HDAC4 knockdown does not appear to have a major effect on the transduction efficiency of the vector, it seems likely that HDAC4 is not required for these steps of the retroviral life-cycle. Nevertheless, it is possible that HDAC4 affects the life-cycle in different ways. One possibility is that HDAC4 has a cell-type-specific function and affects the retroviral life-cycle differently depending on cell type. Another possibility is that HDAC4 affects intracellular or intranuclear trafficking, which may effect integration site selection. Experiments designed to test these hypotheses are underway in our laboratory.

What are the practical implications of our data? HIV-1-based vectors perform integration and PIR in an identical way to wild-type HIV-1. Thus, proteins which are required for PIR of HIV-1-based vectors are also involved in PIR of HIV-1. Since PIR is absolutely required for HIV-1 replication, proteins involved in PIR are potential targets for ani-HIV-1-therapy. However, overlapping functions of these proteins suggest that it will be necessary to inhibit more than one DNA repair pathway to achieve complete suppression of HIV-1 replication.

Finally, our results indicate that HDAC4 accumulates at the sites of integration. HDAC4 foci thus may serve as a useful marker for integration, and their numbers could be used to evaluate the efficacy of HIV-1- inhibitors at the early steps of the HIV-1- life-cycle.

## Experimental Procedures

### Cells

HeLa cells were maintained in DMEM medium supplemented with 10% fetal bovine serum and antibiotics (Penn/Strep).

### HIV-1-based vectors

All VSV G-pseudotyped HIV-1 based vectors were prepared as described previously [33,34], and carried either a *lac*Z or EGFP reporter gene.

### Plasmids and transfections

Plasmids expressing the full-length HDAC4 (amino acids 1-1084) fused to the EGFP protein (HDAC4-1084) or the HDAC4 C-terminal truncated mutant, lacking the nuclear export signal (amino acids 1-1061) fused to the EGFP protein (HDAC4-1061) have been described, and were a generous gift from Dr. X. J. Yang of McGill University [12,29]. The PEGFP-C1 control plasmid expressing the EGFP protein under control of the CMV promoter was purchased from Clontech (GenBank Accession # U55763). Plasmids were transfected into HeLa cells using the Lipofectamine™ 2000 transfection reagent (Invitrogen, cat # 11668-027) using company protocols. Cells were infected with the HIV-1-based vector two days post-transfection.

### Chromatin Immunoprecipitation

HeLa cells were infected at a multiplicity of infection (m.o.i.) 0.1 for the indicated time intervals. In some cases, an integrase inhibitor was added at the time of infection (118-D-24, NIH AIDS Reagent Program). Cells were then harvested and ChIP was performed as described [35], with the anti-HDAC4 antibody (1 μg/sample, Santa Cruz Biotechnology, cat # sc-11418X) or anti-HDAC2 antibody (1 μg/sample, Abcam, cat # ab16032) or anti-HDAC6 antibody (1 μg/sample, Santa Cruz, cat # sc-11420). Protein-associated vector DNA was detected by real-time PCR, using primers and probes detecting HIV-1 LTR. Forward primer: 5'-TGTGTGCCCGTCTGTTGTGT-3'; Reverse primer: 5'-CCTGCGTCGAGAGAGCTC-3'. To quantitate the viral amplicon, a TaqMan dual 5'-6-carboxyfluorescein-and 3'-6-carboxytetramethylrhodamimine-labeled probe was used: 5'-(FAM)-CAGTGGCGCCCGAACAGGGA-(TAMRA)-3' (Integrated DNA Technologies). Real-time PCR was performed using a LightCycler 1.5 with software 3.5.3 (Roche). Reaction mixtures contained QuantiFast Probe 2× mix (Qiagen), 100 nM probe, and 200 nM primers. The standard cycling conditions were 95°C - 3 min followed by 50 cycles at 95°C - 3 s and 60°C - 30 s. Samples were run in triplicate.

### Immunofluorescence experiments

HeLa cells were plated at a density of 2 × 10^4 ^and grown on 4-well chamber slides. The following day, the cells were infected with the HIV-based vector at m.o.i. 10 for a time course study at 4, 6, and 8 hours. In another experiment, vector and an integrase inhibitor (118-D-24, final concentration of 100 μM) or the vector only had been incubated for 8 hours prior to fixation. At the indicated time points, cells were washed in PBS and fixed by adding cold methanol-acetone (1:1 volume) at room temperature for 2 min. The slides were incubated with the primary antibody in KB buffer overnight at 4°C. As a control, we used samples incubated in KB buffer with no primary antibody. The primary antibody was the rabbit polyclonal anti-HDAC4 (see above), diluted 1:500 in KB buffer. The secondary antibody, Alexa Fluor 488 donkey anti-rabbit (Invitrogen, cat # A21206) was used at a 1:1000 dilution. Cells were then washed with PBS containing 0.1% Triton. Cells were incubated in the secondary antibodies for 1 hr at room temperature. Cells were then stained directly with 4',6-diamidino-2-phenylindole, dihydrochloride (DAPI) (Invitrogen, cat # D1306) for 5 min at room temperature. The stained cells were washed with KB buffer and mounted with prolong gold anti-fade (Invitrogen, cat # P36930). Images of stained cells were taken using a Nikon Eclipse TE-2000 S with fluorescence optics at an objective magnification of 20×.

### Quantitation of HDAC4 foci in infected cells

Random images of HeLa cells stained as described above were taken using Nikon Eclipse TE-2000 S at a magnification of 20×. All cells were then counted on a randomly selected slide, both to determine the number of cells containing foci and number of foci per cell, if the cell contained foci. This had been performed in duplicate, each time on a different slide.

### Alu-PCR

To detect and quantify fully integrated proviral DNA, a two-step nested *Alu*-PCR technique was conducted. Cells were infected with the HIV-1-based vector at m.o.i. 0.1. Three days post-infection genomic DNA was extracted (Qiagen, cat # 51306). The first round of *Alu*-PCR employed a primer targeting the cellular *Alu *sequence 5' - GCCTCCCAAAGTGCTGGGATTACAG - 3' as well as the primer targeting the HIV-1 LTR/*gag *region, 5' - TTTTGGCGTACTCACCAGTCG - 3'. This initial amplification step used 100 ng of genomic DNA as template. Samples were subjected to 30 PCR cycles of 95°C - 30 s, 60°C - 45 s, and 72°C - 5 min, and after the final round, samples were kept at 72°C for 10 min. Products of the first round (4 μl of the 50 μl first round reaction) were used in the second, real-time PCR reaction as described above (see ChIP experiments).

### HDAC4 siRNA-mediated knockdown

A pool of siRNAs targeting HDAC4 (cat # M-003497-03) and a pool of non-targeting, control siRNA (cat # D-001206-14-05) were obtained from Dharmacon. A day after plating 10^5 ^HeLa cells per 60 mm dish, cells were transfected with siRNA using Lipofectamine™ RNAiMAX Transfection Reagent (Invitrogen, cat # 13778-075) according to the manufacturer's protocol. The following day, medium was replaced, and cells were transfected again the same way. The next day (three days after cells were plated) cells were infected and assayed for integration, see *Alu*-PCR methods above. HDAC4 levels were measured three days after plating by western blotting with an anti-HDAC4 antibody (cat # sc-11418, Santa Cruz Biotechnology).

### Detection of apoptosis by western blotting

HeLa cells (transfected with either a control C1, HDAC4-1061 or HDAC4-1084 plasmid two days prior to infection, see above) were infected at mo.i. 2. KU-55933 (Calbiochem, cat # 118500-2 MG) was added at the time of infection to a final concentration of 10 μM. One day post-infection, cells were harvested, lysed and cell lysates subjected to western blotting with an anti-PARP antibody (sc-7150, Santa Cruz Biotechnology).

## Competing interests

The authors declare that they have no competing interests.

## Authors' contributions

JAS carried out the HIV-1 transduction experiments and real-time PCR-based assays. JY carried out the immunofluorescence experiments. RD wrote the manuscript and participated in western blotting and ChIP experiments GDK participated in immunofluorescence and transduction experiments. All authors read and approved the final manuscript.
